# Sodium N-lauryl amino acids derived from silk protein can form catanionic aggregates with cytarabine as novel anti-tumor drug delivery systems

**DOI:** 10.1080/10717544.2020.1742250

**Published:** 2020-03-26

**Authors:** Meng Zhang, Shu-Xiang Zhao, Biao Ding, Yu-Qing Zhang

**Affiliations:** a Silk Biotechnology Laboratory, School of Biology and Basic Medical Sciences, Soochow University, Suzhou, China;; b National Engineering Laboratory for Modern Silk, Soochow University, Suzhou, China

**Keywords:** Silk sericin, surfactant, cytarabine, catanionic aggregates, drug delivery

## Abstract

A sodium N-lauryl amino acids (shortly silk sericin surfactant, SSS) is synthesized with lauryl chloride and sericin amino acids recovered from silk industrial waste. The purpose of this study is to explore whether the sericin surfactant can be used as a potential drug delivery carrier. By controlling the proportion of cationic drugs, cytarabine hydrochloride (CH) and anionic SSS, the aggregation behavior, slow release capability and toxicological effects of catanionic aggregates or vesicles, formed through CH and SSS, have been investigated in detail. Dynamic light scattering (DLS), transmission electron microscopy (TEM), and zeta potential analysis showed that the aggregate solution could form a stable vesicle structure when the mass fraction of CH is less than or equal to 0.3. The drug release results showed that the cumulative release rate of the catanionic aggregation solution with CH mass fraction of 0.2 reached a maximum at 18 h, being approximately 9 times greater than that of pure cytarabine. The CH/SSS aggregates had a significant sustained release effect compared with the control group. At the same time, vesicles formed by SSS and CH have better anti-tumor effects compared with the pure drug group. In summary, sericin surfactant from silk industrial waste has a potential use as a drug delivery carrier.

## 1. Introduction

Drug delivery research has received extensive attention to maximize the therapeutic effect of drug compounds in recent decades (Yang et al., 2018). Drug release carriers have been the focus of attention. Many materials have been reported as carriers for drug release, such as biodegradable dendrimers (Huang & Wu, 2018), chitosan (Ahsan et al., 2018), microemulsions (Gibaud & Attivi, 2012), ZnO-based nanomaterials (Huang et al., 2017), polymeric gels (Cook & Brown, 2018), polymer-coated mesoporous ceramics (Callender et al., 2017), and surfactants (Roig et al., 2018). Surfactants as drug delivery carriers have been greatly developed in recent years; it usually plays the role of drug delivery by forming catanionic vesicles. Catanionic surfactant vesicles have been recognized as having several advantages over conventional liposomes: (1) vesicles form spontaneously without the need for additional treatment, (2) they have a longer shelf life than synthetic vesicles (Marques, 2000), and (3) they have lower cytotoxicity compared to synthetic or purified phospholipids. In addition, the size of catanionic vesicles can easily be controlled by adjusting the mixing ratio of the surfactant and drug.

Surfactants have been widely studied due to their excellent properties (Shiojiri et al., 1996; Shiloach & Blankschtein, 1998; Mehling et al., 2007; Ampatzidis et al., 2014). Many types of surfactants can be divided into anionic surfactants, cationic surfactants, amphoteric surfactants, etc. The application of anionic surfactants in biochemistry and drug release has attracted attention because of the lower toxicity compared with other types of surfactants (Murguia et al., 2008; Chaudhari & Dugar, 2017). An N-lauryl amino acid surfactant is a typical anionic surfactant. It not only has the characteristics of low cytotoxicity of traditional surfactants but also has the advantages of easy availability from raw materials and easy degradation (Sanchez et al., 2007; Faustino et al., 2011; Tripathy et al., 2018). The role of amino acid surfactants in antibacterial and solubilization applications has been reported (Coronel-Leon et al., 2017; Hong et al., 2018).

Sericin protein surfactant (SSS) is a sodium N-lauryl amino acids prepared from silk sericin industrial waste. Sericin is needed to be solved in the treatment of silk industrial waste, and the preparation of sericin protein surfactant provides a new solution for silk industry waste. The properties and synthesis of SSS have been studied in detail by our team (Wu & Zhang, 2014; Ding et al., 2017). In this article, for the first time, we describe the formation of catanionic vesicles, formed using the anionic surfactant (SSS) and a cationic drug. Cytarabine hydrochloride was chosen as a model cationic drug to form catanionic vesicles with SSS. The effect of the concentration ratio of SSS to CH on the physicochemical properties of the mixed solution was studied. To better understand the relationship between SSS and CH, systematic research with transmission electron microscopy (TEM), conductivity and so on was performed. The drug release behavior of different proportions of catanionic aggregates was also studied. At the same time, the anti-tumor effect of catanionic aggregates was detected. The purpose of this article is to elucidate the interaction between anionic surfactants prepared from sericin and oppositely charged species and to prove that it can be used as a drug release carrier with the possibility of applying it to the human body.

## 2. Materials and methods

### 2.1. Materials

Cytarabine hydrochloride (CH) was purchased from Suzhou Geruite Medicine Technology Co Ltd. Lauroyl chloride was purchased from Jiangsu Nantong Rudong Lianfeng Chemical Co., Ltd. Other chemical reagents were from the Experimental material supply Center of Soochow University. New fetal bovine serum, trypsin, streptomycin, RPMI 1640 medium, DMEM medium, and double antibody were purchased from Bioengineering (Shanghai) Limited by Share Ltd. Cocoons were provided by Silk Biotechnology Laboratory, Soochow University. Sericin protein surfactant (SSS) was prepared in our laboratory.

### 2.2. Experimental

#### 2.2.1. Preparation of sericin amino acid

According to the method previously reported by the author (Wu & Zhang, 2014), cocoons were degummed with 62.5 mmol/L NaOH solution to obtain alkaline silk degumming solution. Spray drying the obtained sericin solution to obtain crude sericin powder. The sericin powder obtained was dissolved in hydrochloric acid with a concentration of 6 mol/L, and then hydrolyzed at 121 °C for 0.5 h. Adjust pH to about 10, add alkaline protease for enzymatic hydrolysis, and then heat up in boiling water bath to remove alkaline protease. The sericin amino acid powder was obtained by spray drying of sericin amino acid solution, which was used for the synthesis of sericin protein surfactant.

#### 2.2.2. Preparation of sericin protein surfactant

Sericin amino acid powder (50 g) was dissolved in 1 L water, and then the pH of the solution was adjusted to 8.0–9.0. The prepared amino acid solution was placed in the reactor, and 1 L acetone was added to the glass reactor at the same time. The temperature of the reaction system was controlled within 5 °C, and then 99% lauroyl chloride was slowly dripped into the system for 50 mL. The pH value of the whole reaction system was controlled at about 8.0 by dropping 2.0 mol/L sodium hydroxide solution. After dropping, the whole reaction temperature was raised to 20 °C and reacted for 4 hours. Subsequently, the pH value of the mixture was adjusted to 1.0–2.0 with hydrochloric acid of 0.6 mol/L, and the precipitation was the crude product. The obtained precipitate was washed and dried by hot water and hot petroleum ether for several times, in turn, finally, the product was dissolved in ethanol, and the pH of the reaction solution was adjusted to about 7.0–8.0. The reaction continued at room temperature for 30 min, the final product was sericin protein surfactant.

#### 2.2.3. Preparation of catanionic aggregate solution

To prepare the surfactant–drug mixtures, the same concentration of 2.0 mg/mL of anionic surfactant (SSS) and cationic drug (CH) was prepared individually in distilled water and mixed in the desired amount to obtain different mass ratios (1:9, 2:8, 3:7, 4:6, 5:5, 6:4, 7:3, 8:2, and 9:1, *W/W*) of SSS to CH with a total concentration of *C*_total_ = 2.0 mg/mL. *X*_1_ is expressed as the mass fraction of CH (*X*_1_ = *m*_CH_/*m*_(SSS+CH)_). The samples were prepared at 25 °C, and the distilled water was filtered through a 0.45 μm microporous filter membrane in this experiment.

### 2.3. Methods

#### 2.3.1. Transmission electron microscopy (TEM)

The catanionic aggregate solution (SSS-CH) was dripped into the carbon film-copper network to prepare a film at 25 °C. Then, the membranes were quickly inserted into liquid ethane cooled by liquid nitrogen. The samples were dried and finally observed by a JEM-100CXII transmission electron microscope.

#### 2.3.2. Dynamic light scattering

To prepare dust-free solutions for dynamic light scattering, the SSS-CH solutions were filtered through a 0.8 μm membrane filter before measurements. The size of the aggregates was determined by a Nano-ZS90 dynamic light scatterometer (Malvern Inc., UK), with angle detection set at 90°. The samples were located in cuvettes at room temperature, and the cuvettes were washed with distilled water to remove dust.

#### 2.3.3. Turbidity and conductivity measurements

Turbidity measurements were performed with an SGZ-200AS table turbidimeter (Shanghai Yuefeng Instrument and Instrument Co., Ltd., China). The conductivity was measured using a DDS-11A conductivity meter (Shanghai Electric Science Instrument Limited by Share Ltd, China). All measurements were performed at room temperature.

#### 2.3.4. Zeta potential measurement

Zeta potential was determined using a Nano-ZS90 dynamic light scatterometer (Malvern Inc., UK). The samples were located in cuvettes at room temperature.

#### 2.3.5. Drug release experiment

*In vitro* drug release of catanionic mixtures was performed as follows. A certain amount of SSS and CH mixture (*C*_t_ = 2.0 mg/mL) was sealed in a dialysis membrane bag with a molecular weight cutoff of ∼3500 Da and incubated at 37.0 ± 0.1 °C in phosphate buffer solution (pH  =  7.4) for a total volume of 50 mL. The catanionic aggregate solution was continuously stirred at 100 rpm. At selected time intervals, 1 mL of solution was withdrawn from the release media and replaced by the same volume of fresh phosphate-buffered solution (PBS). The release solution was measured with multifunction enzyme-labelled Spectra Max M5 (Molecular Devices Inc., San Jose, CA, USA) at a wavelength of 310 nm, which is a typical absorbance peak for CH. For comparison, the release of pure CH in the release medium was also investigated. The procedure was the same as described above. All operations were carried out in triplicate, and the release of drug was determined by a calibration curve. The cumulative amount of CH released from the catanionic aggregate was calculated using the following equation (*a*%):

$$a\%=\frac{M_{t}}{M_{\text{total}}}\times 100\%$$

where *M*_t_ is the amount of CH released from the catanionic mixture at time *t* and *M*_total_ is the total amount of CH loaded in the catanionic aggregate.

#### 2.3.5. Antitumor activity *in vitro*

Ramous and CEM lymphoma cells were obtained from the Chinese Academy of Sciences, Shanghai, China. The growth medium used was DMEM supplemented with 10% (*v/v*) FBS. The cells were grown in culture dishes (Thermo Scientific, Waltham, MA, USA) at 37 °C in humidified air containing 5% CO_2_.

##### 2.3.5.1. Testing SSS cytotoxicity

SSS cytotoxicity was determined by the CCK8 assay. Ramous and CEM lymphoma cells were incubated with varying concentrations of SSS (*C*_SSS_  =  1, 10, 50, 100, 250 μg/mL). Each condition was repeated six times, and the average was taken. The data were expressed as the mean ± SD and were analyzed using Origin 8.5 software. The cell viability rate in the culture containing the SSS sample was calculated as follows:

$$\text{Cell}\;\text{viability}\;\text{rate}\;(\%)=({\mathrm{OD}}_{\text{sample}}–{\mathrm{OD}}_{\text{blank}})/({\mathrm{OD}}_{\text{control}}–{\mathrm{OD}}_{\text{blank}})\times 100\%$$

In this study, the FBS group was used as the control, and its cell viability was calculated as 100%.

##### 2.3.5.2. SSS-CH antitumor activity

SSS-CH antitumor activity was also determined by the CCK8 assay. The concentrations of SSS-CH (*X*_1_ = 0.2) were 0.1, 1, 10, 50, and 100 μg/mL. Each condition was repeated six times, and the average was taken. The data were expressed as the mean ± SD and were analyzed using Origin 8.5 software. The cell inhibition rate in the culture containing the SSS-CH sample was calculated as follows:

$$\text{Cell}\;\text{inhibition}\;\text{rate}\;(\%)=1–({\mathrm{OD}}_{\text{sample}}–{\mathrm{OD}}_{\text{blank}})/({\mathrm{OD}}_{\text{control}}–{\mathrm{OD}}_{\text{blank}})\times 100\%$$

In this study, the FBS group was used as the control, and its cell viability was calculated as 100%.

##### 2.3.5.3. Microscopic observation

Observation was conducted using an EVOS × 1Core Inverted Microscope (AMG, Bothell, WA, USA) to observe cell morphology in different periods and to acquire images.

## 3. Results

### 3.1. Physicochemical properties of the SSS-CH mixtures

#### 3.1.1. Configuration of SSS-CH aggregation

**Figure 1.**
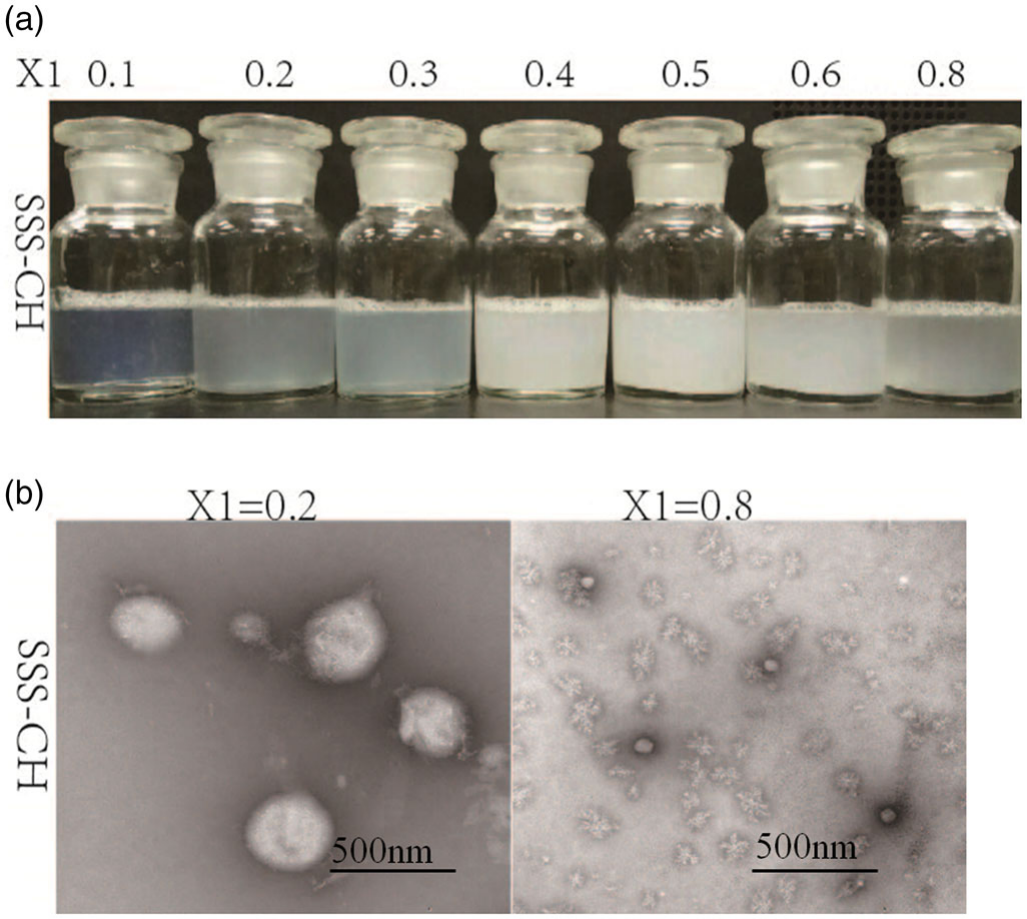
(a) The apparent morphology of catanionic aggregation in solutions of SSS-CH. (b) The TEM photos of SSS-CH catanionic aggregates. *X*_1_ is expressed as the mass fraction of CH (*X*_1_=*m*_CH_/*m*_(SSS+CH)_).

Samples with different concentrations of SSS and CH were prepared at 2.0 mg/mL, and the images of SS-CH catanionic aggregates with different *X*_1_ values are shown in Figure 1(a). The figure shows that the proportion of SSS and CH has a great influence on the morphology of mixed solutions. The samples with *X*_1_<0.4 show a blue or light blue color, inferring the formation of catanionic vesicles in mixed solutions (Thapa et al., 2013; Comelles et al., 2015). In addition, it was discovered that there was turbidity and even precipitate formation, in mixed solutions with *X*_1_≥0.4. Therefore, we speculated that there were no vesicles in the samples with *X*_1_≥0.4.

To verify the above inference, TEM was performed on the catanionic aggregates with *X*_1_ = 0.2 and *X*_1_ = 0.8, as shown in Figure 1(b). The TEM diagram clearly shows the formation of catanionic vesicles in the mixed solution with *X*_1_ = 0.2. The formation of catanionic vesicles is due to the electrostatic interaction between SSS and CH in the mixed solution. However, there is no vesicle formation in the aggregate solution with *X*_1_ = 0.8, and micellar structures are formed. The reason for this is that with the increase in cationic drug concentration, the electrostatic balance between the catanionic vesicles is destroyed, and the vesicle changes into a micellar structure.

#### 3.1.2. Particle size distribution

**Figure 2.**
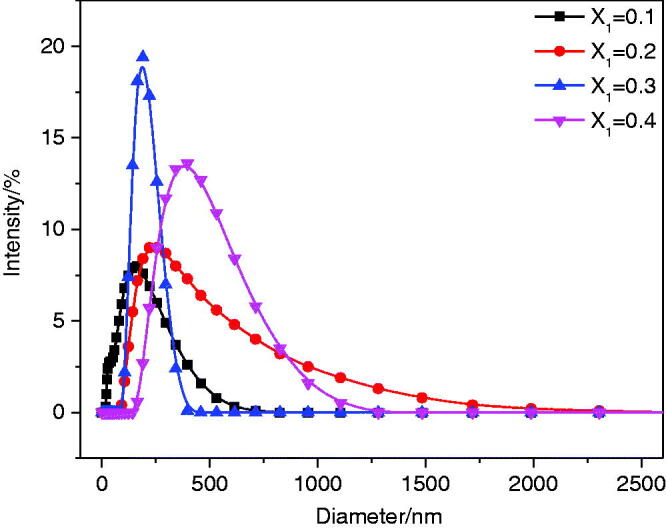
Particle size distribution diagram of SSS-CH catanionic aggregate solutions with different *X*_1_ values.

The size of vesicles plays an important role in the drug delivery system. The DLS experiment was used to measure the particle size of the aggregate solution with *X*_1_≤0.4. The results are shown in Figure 2. The figure shows that the maximum hydrodynamic diameters of the mixed solutions with *X*_1_ = 0.1, 0.2, and 0.3 are 164.2 nm, 232.6 nm, and 190.1 nm, respectively. The maximum hydrodynamic diameter of the sample with *X*_1_ = 0.4 is greater than 400 nm. The change in particle size of the aggregate solution with *X*_1_≤0.4 was speculated as follows. First, with increased concentrations of the cationic drug, anionic surfactants were continuously combined to form catanionic vesicles; thus, the aggregate size increased (0.1<*X*_1_≤0.2). However, as the concentration of the cationic drug continued to increase, the catanionic vesicles rearranged under electrostatic interactions to form smaller vesicles, resulting in a decrease in particle size (0.2<*X*_1_≤0.3). When *X*_1_ = 0.4, precipitation occurred due to the neutralization reaction between surfactant SSS and drug CH.

#### 3.1.3. Conductivity

**Figure 3.**
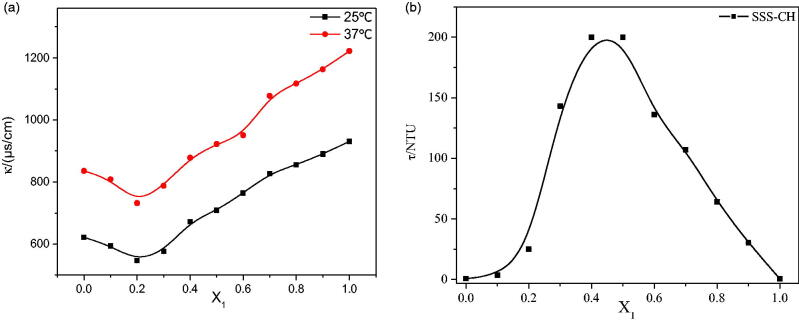
(a) Conductivity diagram of SSS-CH catanionic aggregate solutions with different *X*_1_ values. (b) Turbidity values for SSS-CH catanionic aggregate solutions with different *X*_1_ values.

The conductivity of the aggregate solution at 25 °C and 37 °C is shown in Figure 3(a). The temperature has a significant effect on the conductivity of the aggregate solution, and the conductivity of aggregates at a high temperature is obviously higher than that at a low temperature. The reason for this phenomenon is the accelerated migration rate of ions at high temperatures. At the same time, the change in the conductivity trends for samples at different temperatures is similar. At the same temperature, with an increase in *X*_1_, the conductivity decreases until *X*_1_ = 0.2, and then the conductivity gradually increases until *X*_1_ = 1. The conductivity of the solution is mainly determined by the strength of charged aggregates and free ions. With the constant addition of cationic drugs, the anions in the solution are neutralized to form a vesicle structure, resulting in a decrease in the conductivity of samples with *X*_1_≤0.2. However, the vesicle structure begins to change toward the micelle structure as the cationic drug continues to increase, the particle size decreases, the migration rate increases and the conductivity increases for sample solutions with *X*_1_>0.2.

#### 3.1.4. Turbidity

The turbidity measurement results for mixed solutions with different *X*_1_ are shown in Figure 3(b). As shown in Figure 3(b), the turbidity of the aggregate solution first increases and then decreases, reaching a maximum at *X*_1_ = 0.4 and *X*_1_ = 0.5. The change in turbidity is related to the size and number of aggregates in solution (Goncalves et al., 2007). When *X*_1_≤0.3, the number of vesicles in the aggregate solution gradually increases as *X*_1_ increases, so the turbidity also increases. Maximum turbidity is caused by precipitation of the sample solution with 0.4≤*X*_1_≤0.5. With an increase in *X*_1_, only micelles and drug molecules with smaller particle sizes exist in the mixed solution. Therefore, the turbidity of the aggregate solution with *X*_1_>0.5 decreases.

#### 3.1.5. Zeta potential

Zeta potential is an important index used to measure the stability of vesicle solutions, and it can be used to judge the stability of aggregates (Shen et al., 2009). If there is a high zeta potential in the solution, there will be a strong electrostatic repulsion between particles and no aggregation phenomenon, so it is relatively stable. Conversely, if the zeta potential in the solution is low, the electrostatic repulsion between the particles is low, causing aggregation between the particles, indicating instability. It has been reported that a zeta value of aggregate solutions greater than +30 mV or less than −30 mV is considered to be stable. The zeta potentials of catanionic aggregate solutions with different *X*_1_ are shown in Table 1. From the table, the zeta potential value of the SSS-CH solution increases with increasing *X*_1_. When *X*_1_＝0.1, the zeta potential value of the aggregate solution is −61.7 mV; as *X*_1_ increases to 0.3, the zeta potential value increases to −44.6 mV, which is still less than −30 mV. This indicates that the aggregates formed by SSS and CH can be stabilized in this range. In contrast, when *X*_1_>0.3, the zeta potential value is greater than −30 mV and less than +30 mV; therefore, formed aggregates cannot be stably present. The results show that the ratio of SSS to CH is an important factor which affects the stability of aggregates.

**Table 1.** The zeta potential value of SSS-CH catanionic aggregate solutions with different *X*_1_ values.

*X*_1_	Zeta potential/mV
0.1	−61.7
0.2	−47.8
0.3	−44.6
0.4	−26.5
0.5	−24.8
0.6	−18.8
0.7	−12.9
0.8	−9.4
0.9	−5.7

### 3.2. Drug release *in vitro*

**Figure 4.**
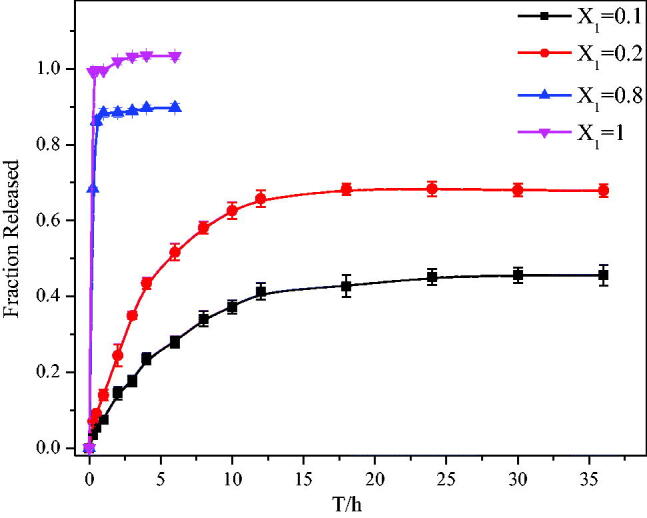
Drug release results for SSS-CH catanionic aggregate solutions with different *X*_1_ values (*n* = 3).

To prove that the prepared SSS-CH aggregates have potential applications for drug delivery, their release behaviors were studied *in vitro*. In this article, phosphate buffer solution (pH  =  7.4) was used as a medium to simulate the blood and fluid environment *in vivo* (Shen et al., 2009). The *in vitro* release behavior of the aggregate solutions with different *X*_1_ was studied under the same drug concentration conditions. Figure 4 shows the drug release curve of the aggregate solutions for *X*_1_ = 0.1, 0.2, 0.8, and 1. It can be seen from Figure 4 that the release rate of the pure drug group is very fast, reaching 100% in 2 h. At the same time, the drug release rates of catanionic aggregates for *X*_1_ = 0.1, 0.2, and 0.8 are 14.45 ± 1.7%, 24.42 ± 2.9%, and 88.48 ± 1.3%, respectively. The cumulative release rate of aggregate solution with *X*_1_ = 0.8 reaches a maximum at 3 h; the maximum release rate is 91.02 ± 1.76%, and it no longer changes in the release rate. However, with the extension of time, the cumulative release rate of catanionic aggregate of *X*_1_ = 0.1 and *X*_1_ = 0.2 continues to increase. The cumulative release rates of catanionic aggregates with *X*_1_ = 0.1 and *X*_1_ = 0.2 reach their maximum at 24 h and 18 h, respectively, and the cumulative release rates are 45.14 ± 2.11% and 68.22 ± 1.49%, respectively. The above results indicate that the aggregate solution does not have a sustained release effect when *X*_1_  =  0.8, while it effectively delays drug release when *X*_1_ = 0.1 and *X*_1_ = 0.2 compared with the pure drug group. This phenomenon is mainly due to the formation of catanionic vesicles in samples with *X*_1_  =  0.1 and *X*_1_  =  0.2, which retards drug release. However, there is no vesicle formation in the sample solution with *X*  =  0.8, so no sustained-release effect is observed. Therefore, at a certain percentage, the surfactant SSS prepared from sericin is a good sustained-release drug carrier and has potential application value.

### 3.3. *In vitro* toxicology test

#### 3.3.1. SSS cytotoxicity detection

**Figure 5.**
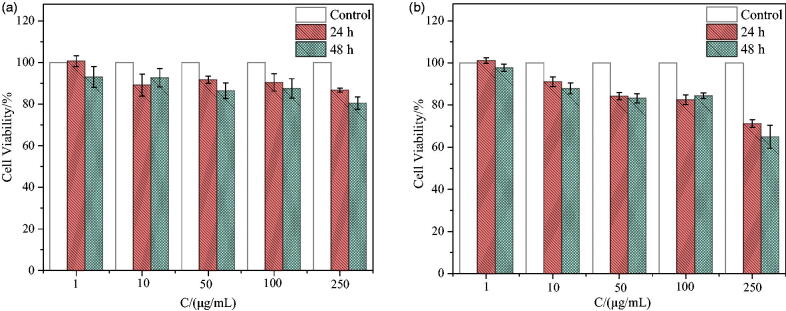
Cell viability in response to different concentrations of SSS incubated with CEM and Ramous cells (*n* = 3). (a, b) Represents the effect of SSS on the survival rate of CEM and Ramous cells, respectively.

As a drug delivery carrier, one of the basic requirements is nontoxicity or low toxicity. The cytotoxicity of synthetic surfactant SSS on two types of lymphoma cells, CEM and Ramous, was evaluated using the CCK8 assay. After incubation for 24 h and 48 h with different concentrations of SSS and two types of cells, the cell survival rates are shown in Figure 5. From Figure 5, the survival rates of CEM and Ramous cell lines are 86.79 ± 0.97% and 71.23 ± 1.85%, respectively, when incubated at the highest concentration of 250 μg/mL for 24 h. After 48 h of incubation, the survival rates of CEM and Ramous cell lines were 80.51 ± 3.00% and 64.98 ± 5.47%, respectively. At a concentration of 100 μg/mL, the survival rate of both cells was greater than 80% after co-incubation for 24 h and 48 h. The prepared surfactant SSS had no obvious cytotoxicity to CEM cells within a certain concentration range.

#### 3.3.2. Anti-tumor research *in vitro*

In this experiment, CEM and Ramous cells were also selected as the research objects of two types of aggregates (*X*_1_ = 0.2), and the anti-tumor effect of pure drug CH was measured as a control. The above studies have shown that when the concentration of surfactant reached 250 μg/mL, it had obvious cytotoxicity to Ramous cells. To eliminate the interference of surfactants, the concentration gradient selected in this experiment was 0.1 μg/mL, 1 μg/mL, 10 μg/mL, 50 μg/mL, and 100 μg/mL. First, the cell morphology of the two cell types was observed under the maximum concentration, and then the inhibitory effects of the samples on the two cell types were determined at different concentrations and times.

##### 3.3.2.1. Observation of cell morphology

**Figure 6.**
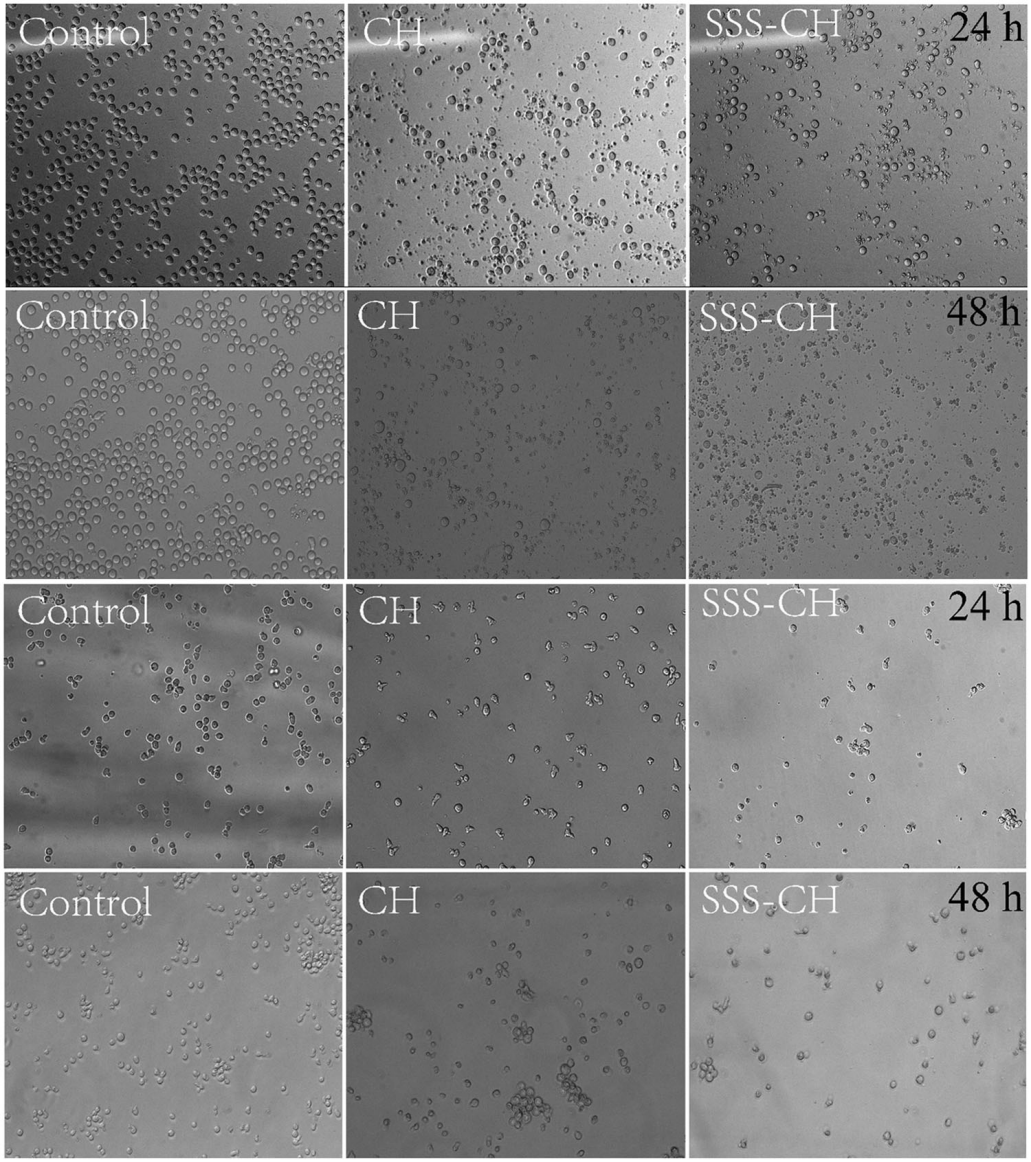
Cell morphologies of CEM (upper) and Ramous (lower) cells incubated with 100 µg/mL samples for 24 h and 48 h. Magnification: ×100.

Figure 6 shows the cell morphology when pure drug CH and aggregate SSS-CH were incubated with CEM and Ramous cells for 24 h and 48 h at the maximum concentration of 100 μg/mL, and the untreated group as the blank control. From Figure 6, it can be found that the cells in the blank control group are clearly outlined and normal in shape, and there is no breakage or shrinkage when they are incubated for 24 h and 48 h. While the cell morphology of the sample group is incomplete, rupture occurs, and the number of cells decreases significantly, indicating that the samples are highly lethal to the two tumor cell types. At the same time, cells incubated with the catanionic aggregate SSS-CH are more severely destroyed compared with the pure drug CH group. The reason may be that the vesicle structure formed in the aggregate solution changes the permeability of the cell membrane to the molecule, and the solute molecule is more likely to enter the cell, thereby enhancing cell death.

##### 3.3.2.2. Inhibitory effect of SSS-CH on CEM cells

**Figure 7.**
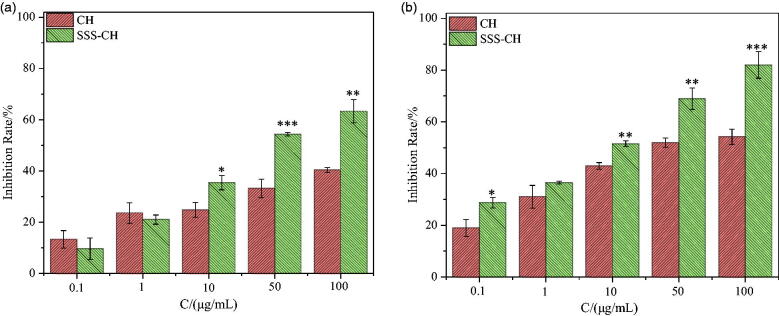
The inhibitory rates of different concentrations of CH and SSS-CH on CEM cells (*n* = 3), (a): 24 h, (b): 48 h, *Indicates the difference compared with the blank group, *p<.05, significant difference, **p<.01, very significant difference, ***p<.001, extremely significant difference.

Figure 7 shows histograms of the cell inhibition rate for different concentrations of the pure drug CH and the aggregate SSS-CH incubated with CEM cells for 24 and 48 h. As we can see, the inhibitory effects of the two types of samples on the CEM cells are significantly dependent on the concentration, and the inhibition rate of the cells increases with the increase of the sample concentrations. At the same concentration, the inhibition rate for the catanionic vesicles is higher than that of the pure drug CH group. As shown in Figure 7(a), the cell inhibition rates for the pure drug CH group and the SSS-CH group are 40.36 ± 0.94% and 63.28 ± 4.55%, respectively, when the concentration of the sample is 100 μg/mL and incubated for 24 h. As shown in Figure 7(b), after 48 h of co-incubation, the cell inhibition rates for the pure drug group CH and SSS-CH are 54.19 ± 2.89% and 82.01 ± 5.15%, respectively. The results show that the formation of vesicles by drugs and surfactants has a stronger toxic effect on CEM cells compared with the pure drug group and has a stronger inhibitory effect on tumors.

##### 3.3.2.3. Inhibitory effect of SSS-CH on Ramous cells

**Figure 8.**
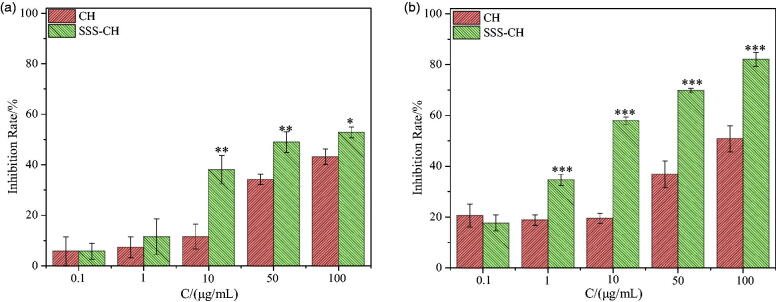
Inhibitory rates of different concentrations of CH and SSS-CH on Ramous cells (*n* = 3). (a): 24 h, (b): 48 h. *Indicates the difference compared with the blank group, *p<.05, significant difference, **p<.01, very significant difference, ***p<.001, extremely significant difference.

Different concentrations of two samples were incubated with Ramous cells for 24 h and 48 h, and the inhibition rate of Ramous cells is shown in Figure 8. Similar to CEM cells, the inhibitory effect of the sample on Ramous cells has a significant concentration and time effect. As the concentration of the sample and incubation time increase, the inhibition rate of the Ramous cells also increases. From Figure 8(a), it can be seen that the cell inhibition rates of the CH group and the SSS-CH group are 43.17 ± 3.05% and 52.82 ± 2.13%, respectively, when the concentration of the sample is 100 μg/mL and incubated for 24 h together. When incubated together for 48 h, the inhibition rates of cells in the CH and SSS-CH groups were 50.85 ± 5.10% and 82.09 ± 2.69%, respectively, as shown in Figure 8(b). We can find that with the extension of culture time, the inhibition rate of SSS-CH groups on tumor cells increased. The growth inhibition of tumor cells in SSS-CH group was better than that in pure drug group *in vitro*, which indicated that SSS-CH group had potentially better tumor inhibition effect.

## 4. Discussion

In recent decades, research on drug delivery systems has become one of the most popular research fields (Uhrich et al., 1999). These drug delivery systems have received widespread attention mainly because of their advantages in improving the efficacy of drugs, reducing the toxicity of drugs, and improving patient compliance and convenience. Drug carriers play an important role in drug delivery systems. Currently, some synthetic polymers are usually used as carriers in drug release systems, but these polymers are usually poor in biocompatibility and have a certain degree of toxicity (Luo & Zai-Jun, 2006). Therefore, the development of high-performance, nontoxic, and sustained-release drug carriers has been very necessary (Kataoka et al., 1993; Lian & Ho, 2001).

Amino acid surfactants are environmentally friendly surfactants that are prepared from biological materials. In addition to their excellent foaming properties and enhanced liquid emulsifying ability, they also have the advantages of low toxicity, good biodegradability, biocompatibility, etc. Therefore, amino acid surfactants have been widely applied to daily chemical products, agriculture, biochemistry, and so on. The application of traditional surfactants for drug release has been reported. However, there are few reports on the application of amino acid surfactants for drug release (Lawrence, 1994; Wong et al., 2006). In the present study, the amino acid surfactant SSS, prepared from silk sericin industrial waste, was used as a sustained-release drug carrier, and the physical and chemical properties, the slow release efficiency and the toxicological effect of the formed aggregates were systematically analyzed.

In this article, it can be observed from the apparent morphology of the catanionic aggregate that when *X*_1_≤0.3, the catanionic aggregate solution exhibits a blue color or a light blue color; that is, the catanionic aggregate has a vesicular structure in this range, and the results of TEM also confirm this speculation. According to the above results, aggregates formed by SSS and drug CH can form vesicle structures and stabilize in a certain range. To determine the sustained drug release ability of the surfactant as a carrier, an *in vitro* sustained release experiment was conducted. The aggregate solution formed by surfactants and drugs does not have a sustained-release effect in a high proportion, but it has an extremely good slow release effect at a lower proportion (*X*_1_ = 0.2). However, we can find that the release behavior of *X*_1_ = 0.1 and *X*_1_ = 0.2 is similar, the error of *X*_1_ = 0.1 at 18 h is too large, which may lead to the delay of accumulation release to 24 h. In fact, the time points at which the two concentrations reach the maximum are similar, about 18 h, both *X*_1_ = 0.1 and *X*_1_ = 0.2 can effectively delays drug release. Considering that the drug will be ultimately applied to the human body, the sustained-release carrier should be characterized as having little to no toxicity. Therefore, we conducted an *in vitro* toxicology study. Within a certain concentration range, SSS has no cytotoxicity. At the same time, with the extension of culture time, the inhibition rate of SSS-CH groups on tumor cells increased. The growth inhibition of tumor cells in SSS-CH group was better than that in pure drug group *in vitro*, which indicated that aggregates formed by SSS and CH had potentially better tumor inhibition effect.

## 5. Conclusion

In summary, the amino acid surfactant SSS prepared from silk sericin industrial waste has stable performance and a good sustained-release effect as a carrier of anticancer drugs, which provides a new potential carrier for the sustained-release and drug effect enhancement of anticancer drugs, provides a new application example and experimental basis for the research of anticancer drugs and surfactants, and provides new hope for the high-value utilization of silk industry waste and the sustainable development of the silk industry.

## Data Availability

The datasets used and/or analyzed during the current study as well as analysis scripts are available from the corresponding author on reasonable request.
